# Excited State Proton Transfers in Hybrid Compound Based on Indoline Spiropyran of the Coumarin Type and Azomethinocoumarin in the Presence of Metal Ions

**DOI:** 10.3390/molecules26226894

**Published:** 2021-11-16

**Authors:** Natalia L. Zaichenko, Tatyana M. Valova, Olga V. Venidiktova, Alexander V. Lyubimov, Andrey I. Shienok, Liubov S. Koltsova, Anton O. Ayt, Galina V. Lyubimova, Leonid D. Popov, Valery A. Barachevsky

**Affiliations:** 1 N.N. Semenov FRC of Chemical Physics of the Russian Academy of Sciences, Kosygin Str. 4, 119991 Moscow, Russia; aleksanlyubimov@yandex.ru (A.V.L.); ashy47@yandex.ru (A.I.S.); koltso50@mail.ru (L.S.K.); galina.lyubimowa@yandex.ru (G.V.L.); 2 Photochemistry Center FSRC “Crystallography and Photonics” of the Russian Academy of Sciences, Novatorov Str. 7a/1, 119421 Moscow, Russia; tatv.photonics@mail.ru (T.M.V.); wolga.photonics@inbox.ru (O.V.V.); ao_ait@mail.ru (A.O.A.); barva@photonics.ru (V.A.B.); 3 Chemistry Department, Southern Federal University, B. Sadovaya Str. 105, 344104 Rostov-on-Don, Russia; ldpopov@mail.ru; 4 Interdepartmental Center of Analytical Research of the Russian Academy of Sciences, Profsoyusnaya Str. 65/6, 117997 Moscow, Russia

**Keywords:** fluorescence, photochromism, spiropyran, azomethinocoumarin, excited state proton transfer

## Abstract

Spectral-luminescence properties of a hybrid compound containing a coumarin-type spiropyran and an azomethinocoumarin fragment in toluene-acetonitrile solution in the presence of Li^+^, Ca^2+^, Zn^2+^ and Mg^2+^ ions are reported. Two excited state proton transfers can occur in the hybrid compound—the transfer of a proton from the OH group of the 7-hydroxy coumarin tautomer to the N atom of the C=N bond of the azomethine fragment leading to green ESIPT fluorescence with a maximum at 540 nm and from the OH group of the 7-hydroxy coumarin tautomer to the carbonyl group of the pyrone chromophore, which leads to the formation of the 2-hydroxyl-tautomer **T** of coumarin with blue fluorescence with a maximum at 475 nm. Dependence of these excited state proton transfers on the metal nature and irradiation with an external UV source is discussed.

## 1. Introduction

Compounds that combine some different photosensitive fragments in one molecule are of great interest, since various combinations of responses (including fluorescence) can be expected in them, which is controlled by the excitation wavelength and the medium (solvent, polymer nature, addition of metal ions). ESIPT systems (systems with intramolecular proton transfer in the excited state) represent one of the fragments often used for introducing in such complicated hybrid compounds, because they demonstrate fluorescence from the enol and/or *cis*-keto forms depending on substituents, geometry, media and excitation wavelength [1,2,3,4,5]. ESIPT-based systems have attracted attention as fluorescent sensors, bio-imaging agents and pH probes. Another important component of complex hybrid photoactive molecules are photochromes [6,7,8], which also have a wide range of possible applications, such as optical molecular memory units, light-transforming membranes, sensors, etc. [9,10,11,12,13,14,15]. We have synthesized a hybrid compound whose molecule is built from photochromic spiropyran fragments and two fluorophores, 7-hydroxycoumarin and azomethin [16]. The presence of a spiropyran fragment in the molecule can lead to photochromic properties due to the spiro-bond cleavage and the formation of a merocyanine form; and 7-hydroxycoumarin and azomethine fragments should provide luminescent properties.

**Scheme 1:**
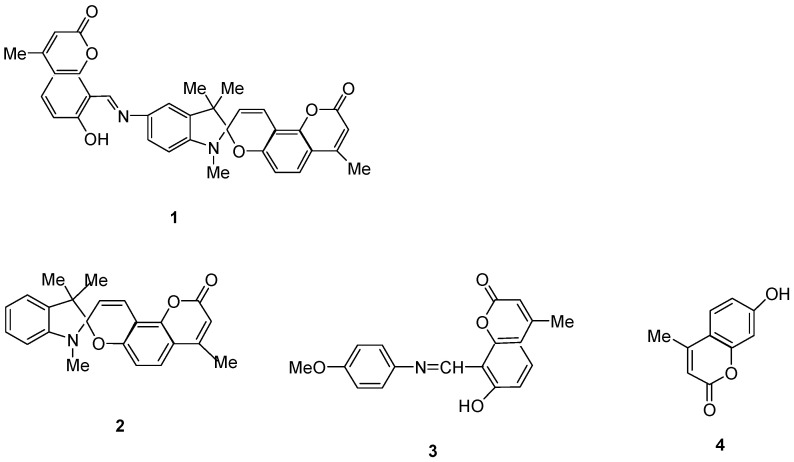
Chemical structures of compounds under study.

Herein dependence of its spectral-luminescent properties in solutions on the metals nature, the excitation wavelength and external UV irradiation is described. Under UV irradiation spiropyrans isomerize to the more polar open merocyanine form. Metal ions can form complexes with the merocyanine molecules, thereby influencing this isomerization process [17]. Subsequent irradiation with visible light results in the formation of the initial closed form, releasing free metal ions. It is therefore possible to trigger metal ion binding by UV irradiation and to reverse this process through visible light irradiation of the colored complex. Moreover, the phenomenon of negative photochromism was observed for a number of complexes formed by the interaction of spiropyran molecules with metal ions [18,19]. At the same time, it should be noted that azomethines are good complexons for metal ions, too [20,21,22,23,24,25]. Thus, in hybrid compound **1** we can expect two centers of complexation—the merocyanine form of the spiropyran fragment and the azomethinocoumarin form. To clarify the nature of the observed absorption and fluorescent properties of hybrid compound **1**, a similar study of model compounds—coumarin spiropyran **2** and azomethinocoumarin **3** was performed. The synthesis of these compounds is described in [16]. The structures of the studied compounds are presented in Scheme 1.

It should be specially noted that we have studied photoinduced dynamic complexes without their separation.

## 2. Results

### 2.1. Design and Synthesis

The synthesis of the hybrid compound 5′-[(7-hydroxy-4-methyl-2-oxo-3*H*-1-benzopyran-8-yl]-1′,3′-dihydro-1′,3′,3′,4-tetramethylspiro[[*2H*,*8H*]benzo[1,2-b,3,4-b’]-dipyran-8,2′-[*2H*]indole]-2-one] **1** and the model 7-hydroxy-8-(4-methoxyphenyliminomethyl)-4-methyl-1-benzopyran-2-one **3** was described earlier [16]. 1′,3′-Dihydro-1′,3′,3′,4-tetramethylspiro[[*2H*,*8H*]-benzo[1,2-b,3,4-b’]-dipyran-8,2′-[*2H*]indole-2-one] **2** was synthesized according to ref. [26].

### 2.2. Abbreviations

The following symbols are used for marking the state of the hybrid molecules components. The capital letters **A** and **B** designate the initial closed and the final open merocyanine fragment states of spiropyran, respectively. The letters with uppercase indexes **E^C^**, **K^C^** and **K^t^** denote the *cis*-enol, *cis*-keto and trans-keto states of the hydroxyazomethine fragment, respectively. The symbol **T** corresponds to the tautomeric 2-hydroxy-form of coumarin. The same abbreviations are used for the corresponding model compounds.

### 2.3. Spectral-Luminescent Properties of Model Compounds

The absorption and fluorescence spectra of the model compounds **2**–**4** in the presence of metal ions are required for the interpretation of complex behavior of the hybrid compound **1**.

**Scheme 2:**
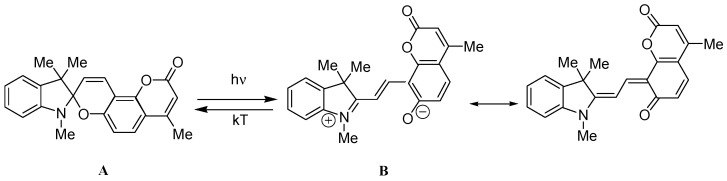
Photochromic processes in spiropyran **2**.

**Figure 1:**
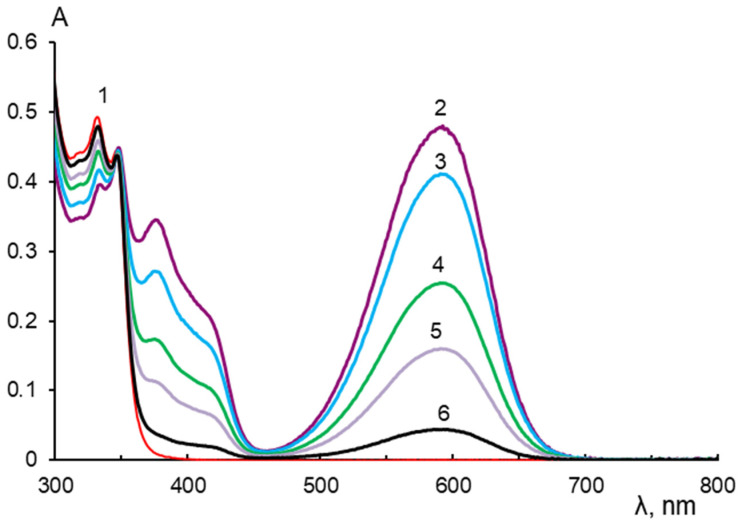
Absorption spectra of compound **2** in the mixed solvent solution before (1), after UV irradiation (2) and after relaxation in the dark (3–6).

The model spiropyran **2** shows photochromic properties typical for spiropyrans (Scheme 2, Figure 1). In mixed toluene:acetonitrile (1:1 by volume) solution, compound **2** exists in the closed colorless form **A,** which absorbs in near UV (<370 nm). Under UV irradiation, the initial colorless form **A** turns into the colored merocyanine form **B** (with a long-wavelength absorption maximum at 600 nm, a short-wavelength absorption maximum at 390 nm and a shoulder at 420 nm (Figure 1) via the O—C_spiro_ bond cleavage, a sequence of rotations and *cis*-*trans* isomerizations. In the dark, the photoinduced bands disappear and the merocyanine form of **2** relaxes to its original colorless state **A** (its photochromic reversibility is presented in Figure S1, Supplementary Materials).

**Scheme 3:**
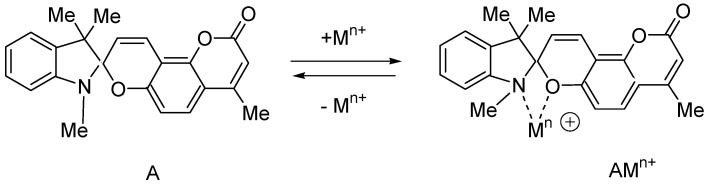
Expected complexation of cyclic form **A** of spiropyran **2.** with metal ions.

We have studied the complex formation in solutions of compound **2** with Li^+^, Ca^2+^, Zn^2+^ and Mg^2+^ ions. Most of the studied complexes are characterized by low efficiency of photochromic transformations, which can be seen from the values of ΔD_B_^phot^ (Table S1). To explain these results, the following Scheme 3 of complex formation between spirocyclic form **A** and metal ions can be proposed as a hypothesis.

**Scheme 4:**
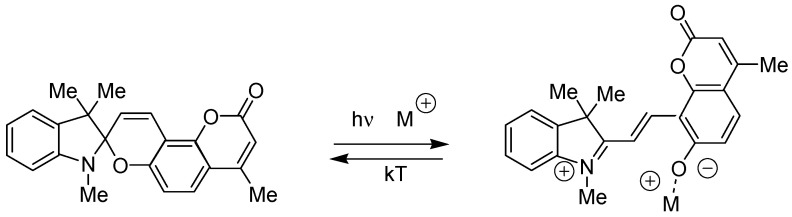
Photoinduced complexation of the ground state of spiropyran **2** form **B**.

These complexes (**AM^n+^**) could not be detected spectrally straightforward, but the decrease in the efficiency of photoinduced spirocycle opening indicates the possibility of their formation. Consequently, after an addition of metal ions into the solution we have an equilibrium between uncomplexed molecules of **A** and its complexes (with metal ions (**AM^n+^**), and only uncomplexed molecules of **A** of spiropyran **2** participate in photochromic transformations. These free molecules can produce photoinduced complexes of the phenolate oxygen atom of the ground state of merocyanine form **B** with metal ions, as evidenced by the hypsochromic shift of its absorption maximum in the presence of metal ions (Table S1), typical for indoline nitrosubstituted spiropyrans (Scheme 4) [18,27].

The photochromic properties of compound **2** are strongly manifested only in the presence of Li^+^ cations (Figure S2) possibly due to low complexing properties of Li^+^. The fluorescent properties of the merocyanine complexes are weak due to the low efficiency of the photochromic reactions.

**Scheme 5:**
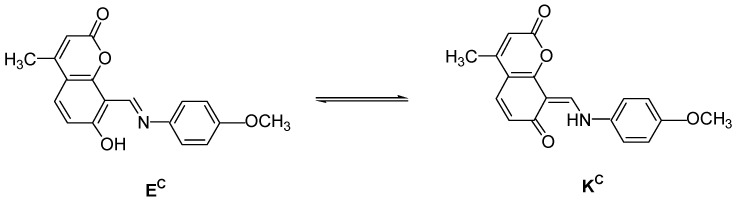
Tautomeric forms of the model compound **3**.

The model compound **3** represents coumarin **4** with an azomethine fragment, which in common corresponds to a fragment of hybrid compound **1**. Azomethinocoumarin molecules **3** as all anil-type compounds may exist in **E^C^** and **K^C^** tautomeric forms (Scheme 5) depending on the environmental conditions [28,29,30].

**Figure 2:**
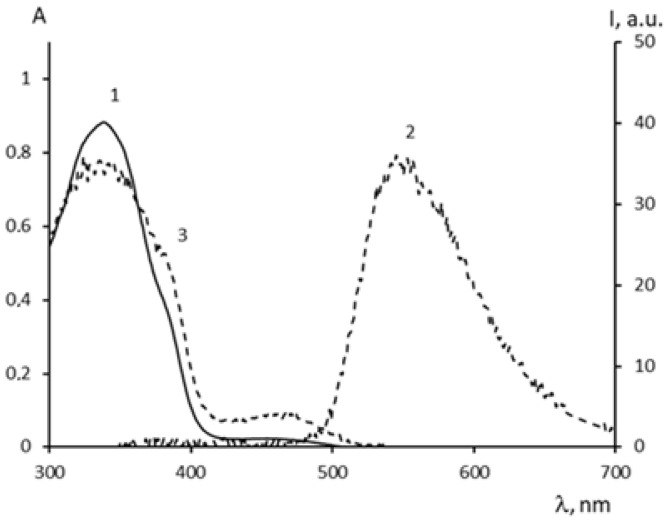
Absorption spectrum (1), fluorescence excitation spectrum at λ_reg_ = 549 nm (3) and fluorescence spectrum at λ_ex_ = 338 nm (2) of compound **3** in the mixed solvent solution.

**Scheme 6:**
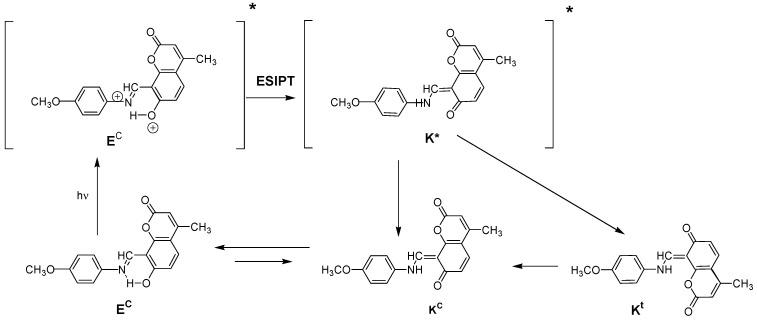
Photoinduced transformations of the model compound **3** under UV irradiation.

The **E^C^** form of anil-type compounds absorbs in near UV and is usually more stable in aprotic solvents. The **K^C^** form has a characteristic absorption band with a maximum near 450 nm [16]. According to spectral characteristics (Table 1, Figure 2), the model compound **3** in the mixed solvent exists for the most part in **E^C^** form (with a maximum at 340 nm), although the presence of very small amount of **K^C^** form (with a maximum at 457 nm) is observed. Only a single fluorescence band with a maximum at 545 nm is observed for **3** (Figure 2, spectrum 2). The fluorescence excitation spectrum of this compound is in good agreement with its absorption spectrum. According to the large value of the Stokes shift (≈11,000 cm^−1^), this emission is caused by excited state proton transfer (ESIPT) in azomethinocoumarin fragment according to Scheme 6.

**Figure 3:**
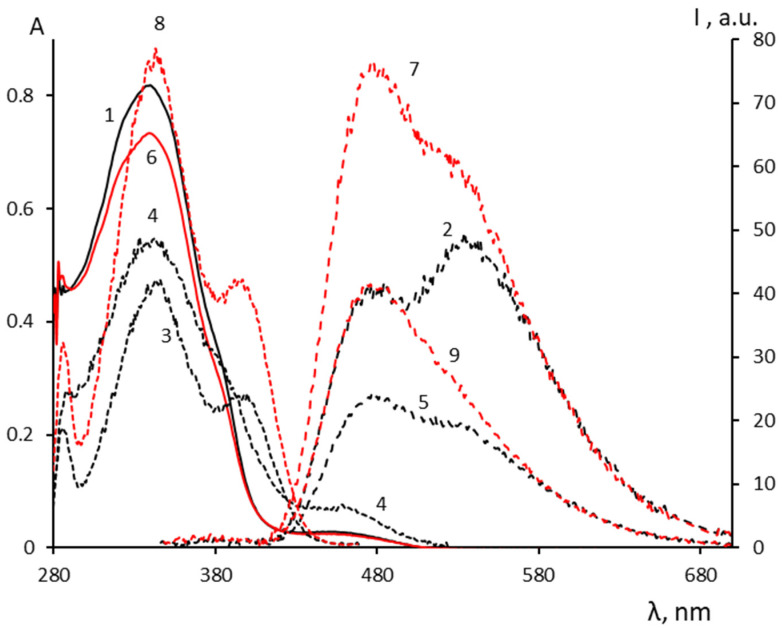
Absorption spectra (1, 6), fluorescence excitation spectra at λ_reg_ = 477 nm (3, 8), 534 nm (4) and fluorescence spectra at λ_ex_ = 338 nm (2, 7) and 399 nm (5, 9) of model **3** in the mixed solvent solution in the presence of Mg^2+^ ions before (1–5) and after UV irradiation with the external source (6–9).

Addition of Mg^2+^ ions to the solution of **3** leads to changes in its absorption and fluorescence spectra (Figure 3, Table 1) contrary to the situation with addition of Li^+^ and Ca^2+^ ions which have no influence on the spectral-luminescent properties of **3** (Table 1).

**Figure 4:**
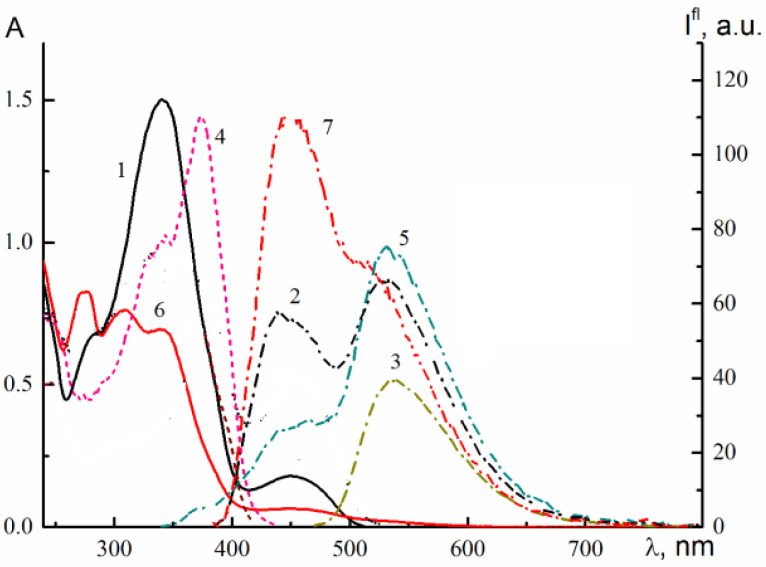
Absorption spectra (1, 6), fluorescence spectra at λ_ex_ = 332 nm (5), 375 nm (2, 7), 461 nm (3) and fluorescence excitation spectra at λ_reg_ = 461 nm (4) before (1–5) and after (6, 7) UV irradiation by the external source of model **3** in ethanol solution. Reproduced from Ref. [16] with permission from the Royal Society of Chemistry.

A comparison of the fluorescence spectra of **3** in the mixed solvent solution in the presence of Mg^2+^ ions (Figure 3, spectra 2, 5, 7) with its spectra in an ethanol solution without metal ions (Figure 4, spectra 2, 5, 7) demonstrates their close similarity in the shape of the spectra and dependence on the excitation wavelength [5].

In both cases two emission bands (with maxima at 478 nm and 534 nm in the mixed solvent solution (Figure 3, spectrum 2) and at 461 nm and 530 nm in ethanol (Figure 4, spectrum 5) are observed at the excitation at the absorption maxima, and the long-wavelength emission predominates in both cases. An increase in the excitation light wavelength leads to an increase in the short-wavelength emission (Figure 3, spectrum 5 and Figure 4, spectrum 2). Only one emission with a maximum at 530 nm is observed at 461 nm excitation (Figure 4, spectrum 3) in ethanol, that is, at the direct excitation of *cis*-keto form of the azomethine fragment having an absorption maximum at 450 nm [26]. The same is observed in the mixed solvent solution in the presence of Mg^2+^ ions.

According to the large value of the Stokes shift (18,000 cm^−1^ in mixed solution and 11,000 cm^−1^ in ethanol) the long-wavelength emission is ascribed to fluorescence of K^c*^ isomer of **3** formed as a result of ESIPT coupled with the isomerization according to Scheme 6. This ascription is also confirmed by the direct excitation of *cis*-keto form described above (Figure 4, spectrum 3).

**Scheme 7:**
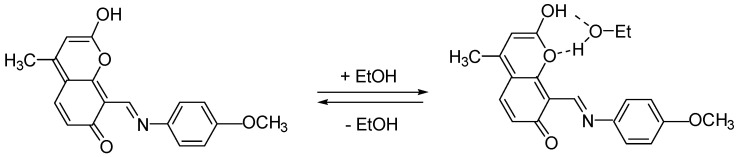
Expected scheme of the associate formation of model **3** with ethanol.

After UV irradiation by an external source, the intensity of short-wavelength fluorescence increases sharply (Figure 3, spectra 8, 10; Figure 4, spectrum 7) and it prevails at any excitation wavelength. It follows from these data that in both cases considered above, the same form of model **3** fluoresces in the short-wavelength region (with a Stokes shift = 7600 cm^−1^), which is stabilized by the formation of an associate of the pyrone fragment with EtOH in ethanol solution according to Scheme 7, and with Mg^2+^ complex formation in the latter case.

**Scheme 8:**
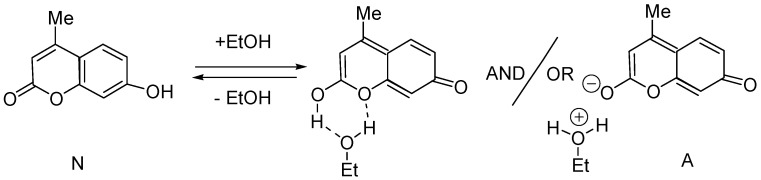
Expected scheme of an associate formation of parent coumarin **4** with ethanol.

Earlier [16] we proposed for model **3** the following scheme of an associate formation with ethanol which is similar to association of the parent coumarin **4** with ethanol (Scheme 8). This associate with ethanol is characterized by the absorption band with a maximum at 373 nm. It should be taken into account that the associates of coumarin **4** and model **3** with ethanol are formed due to hydrogen bonding interaction, because sufficiently stable six-membered systems are formed in this case.

**Scheme 9:**
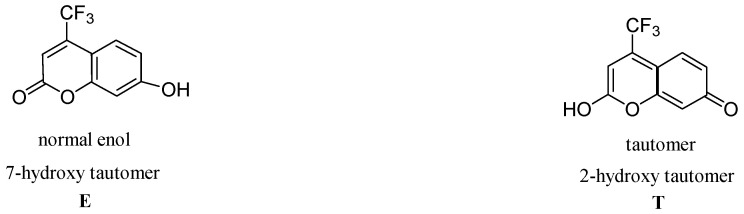
Tautomeric forms of 4-trifluoro-7-hydroxycoumarin.

According to literature data [31,32], there are two tautomeric forms of 4-trifluoromethyl-7-hydroxycoumarin (Scheme 9) in neutral media: normal 7–hydroxy isomer (**E**) and the 2–hydroxy tautomer (**T**).

Form **T** becomes predominant in the excited state of the molecule, because in this form the electron density is transferred from the 7-hydroxyl group to the carbonyl group [31,32,33]. The key factors in the formation of the tautomeric form **T** are protic solvents, such as water, which can give a proton to the carbonyl in the excited state, and UV irradiation, which converts the molecule to the excited state. This form is not registered in the absorption spectra, but is manifested in the luminescence spectra. In hydroxylic solvents such as ethanol, there is a possibility to convert N* directly to T* by means of the concerted hydrogen transfer from the hydroxyl site to the carbonyl site by means of hydrogen bonds of solvent molecules [34]. As it was shown in this work, the band of T* appears distinctly in the nonhydroxylic solvents upon the addition of a small amount of H_2_O.

**Scheme 10:**
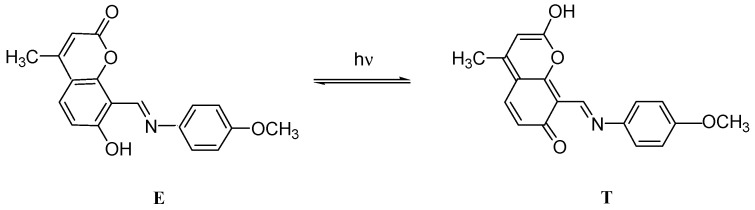
Formation of the tautomeric form **T** of compound **3**.

We can see from the Scheme 8, that association of 7-hydroxycoumarin with ethanol leads to tautomeric form **T** with **e**mission at 447 nm [16]. It can be assumed that in the case of compound **3,** similarly to coumarin **4** [16], there can be also two tautomeric structures of the coumarin part (Scheme 10) and only one of them, corresponding to the form **T**, can give an associate with ethanol. Since the fraction of this structure is small, the portion of the associates is small too.

**Scheme 11:**
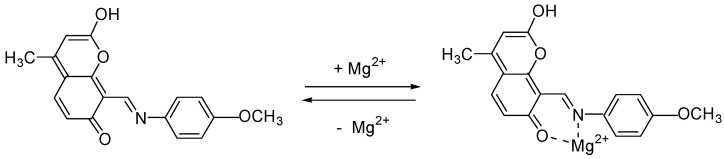
Complexation of model **3** with Mg^2+^ ions.

Consequently, it is reasonable to propose a corresponding structure of a complex of **3** with Mg^2+^ ions (Scheme 11).

In the case of interaction with Mg^2+^ ions, association due to hydrogen bonds cannot occur, and therefore the formation of a six-membered chelate complex of a salt-like type with the tautomeric form **T** of the coumarin fragment (Scheme 11) should be proposed. This assignment coincides with the literature data on the fluorescence of azomethinocoumarin complexes with Mg^2+^ ions and other metals, observed in the region of 470–490 nm [20,21,25].

DFT calculations of both forms were produced to answer the question: which complex of two tautometric forms of azomethinocoumarin with Mg^2+^ ions is more stable? (Figure S3). The calculations were produced for molecules ground state taking into account a solvent. For the used mixture of solvents we assumed ε = 19, where ε-dielectric constant. Calculation results showed that energy of **T** form in a ground state is lower by 0.082 eV than energy of **E** form one. That means that equilibrium between these two tautometric forms is shifted to the **T** form complex, which is significantly more stable than **E** form one. This significant sustainability of the **T-**form one is defined by a higher value of partial charge of Mg atom that is +1.74 in comparison to +1.67 of Mg atom for **E**-form complex. Corresponding bond distances Mg^2+^-N and Mg^2+^-O are 2.13 Å and 1.91 Å for the **E**-form complex and 2.09 Å and 2.05 Å for the **T**-form one.

When Zn^2+^ ions are added to the solution of compound **3**, similar phenomena are observed, but less pronounced (Table 2, Figure S4), that is, a similar complexation can be assumed there too.

### 2.4. Spectral-Luminescent Properties of the Hybrid Compound ***1***

**Figure 5:**
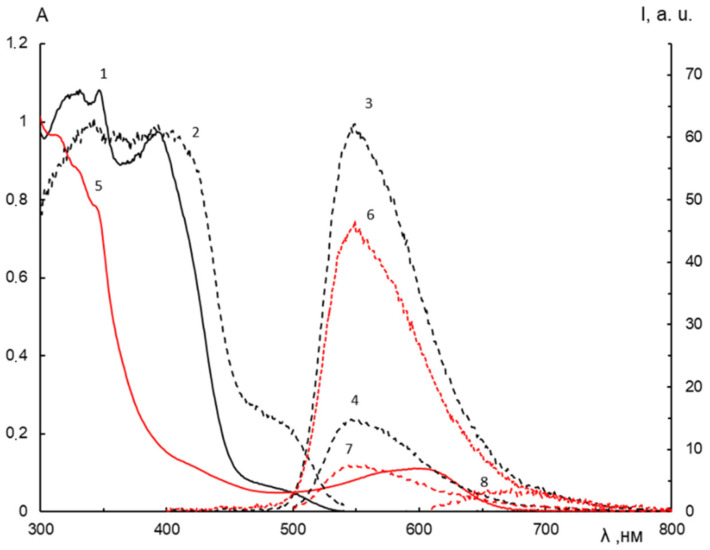
Absorption spectra (1, 5), fluorescence excitation spectrum at λ_reg_ = 550 nm (2) and fluorescence spectra at λ_ex_ = 390 nm (3, 6), 490 nm (4, 7) and 600 nm (8) of compound **1** before (1–4) and after (5–8) UV irradiation by the external source (5–8) in the mixed solvent solution.

**Scheme 12:**
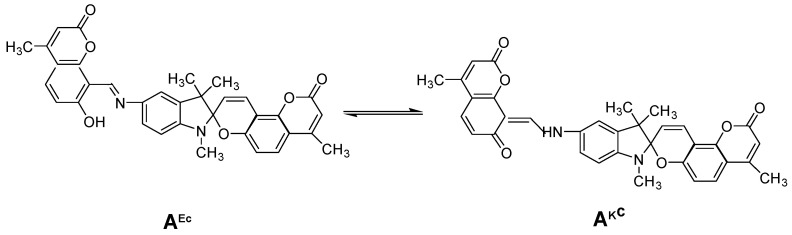
Tautomeric equilibrium between the **A^Ec^** and **A^Kc^** forms of the hybrid compound **1**.

It is evident from the absorption spectra that in the mixed solvent solution compound **1** exists mainly in the **A^Ec^** form being characterized by several absorption bands in the short-wavelength spectral region (<450 nm) (Figure 5, spectrum 1; Table 2). A small amount of **A^Kc^** form with an absorption band at 475 nm exists in equilibrium with the **A^Ec^** form (Scheme 12).

**Scheme 13:**
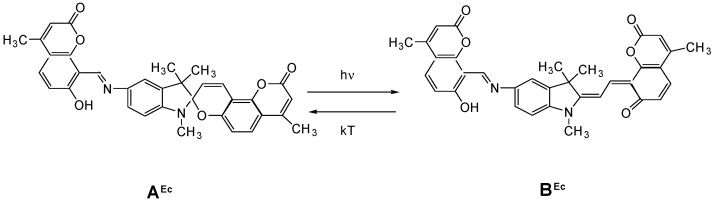
Photochromic transformations of the hybrid compound **1**.

Compound **1** demonstrates photochromic properties typical for spiropyrans (Scheme 13). After UV irradiation a new broad absorption band in the spectral region of 500–700 nm appears (Figure 5, spectrum 5) which is typical for the open merocyanine form **B**, formed via the C_spiro_-O bond cleavage and a sequence of rotations and *cis*-*trans* isomerizations. This photoinduced absorption band disappears reversibly after switching off the activating UV irradiation (its photochromic reversibility is presented in Figure S5). It should be noted that the time of irradiation of the solutions with an external UV source during spectral-luminescence studies does not exceed 30 s, which is far less than the time of the beginning of their photodegradation (see Figures S1 and S5).

The initial **A^Ec^** form of **1** is characterized by one fluorescence band with a maximum at 550 nm (Figure 5, spectrum 3). On the basis of the data for model compounds we can attribute it to the fluorescence of the excited *cis*-ketone A^Kc*^ formed in the process of ESIPT in the azomethinocoumarin fragment. The photoinduced form **B^Ec^** has an emission maximum at 665 nm (Figure 5, spectrum 8). When it appears, the intensity of the fluorescence band of the **A^Ec^** form at 545 nm decreases (Figure 5, spectrum 7).

Introduction of metal ions into the solution of **1** even in the dark leads to a raise of absorption in the 400–500 nm region and an appearance of the absorption band in the 540–580 nm region (Table 2), but these changes are not significant in all cases except Mg^2+^ and Zn^2+^ ions. This indicates that, like compound **3** (Scheme 9), the hybrid compound **1** may form complexes with metal ions via chelation by the azomethine moiety (λ_max_ ≈ 470 nm) and through phenolate oxygen complexation of the merocyanine form **B^Ec^** (λ_max_ ≈ 540–580 nm). The fluorescent properties of the compound **1** change insignificantly after addition of Li^+^ (Figure S6) and Ca^2+^ ions (Figure S7), whereas significant changes appear in the presence of Mg^2+^ ions and to a smaller extent in the presence of Zn^2+^ ions (see Figure S8, Table 3).

**Figure 6:**
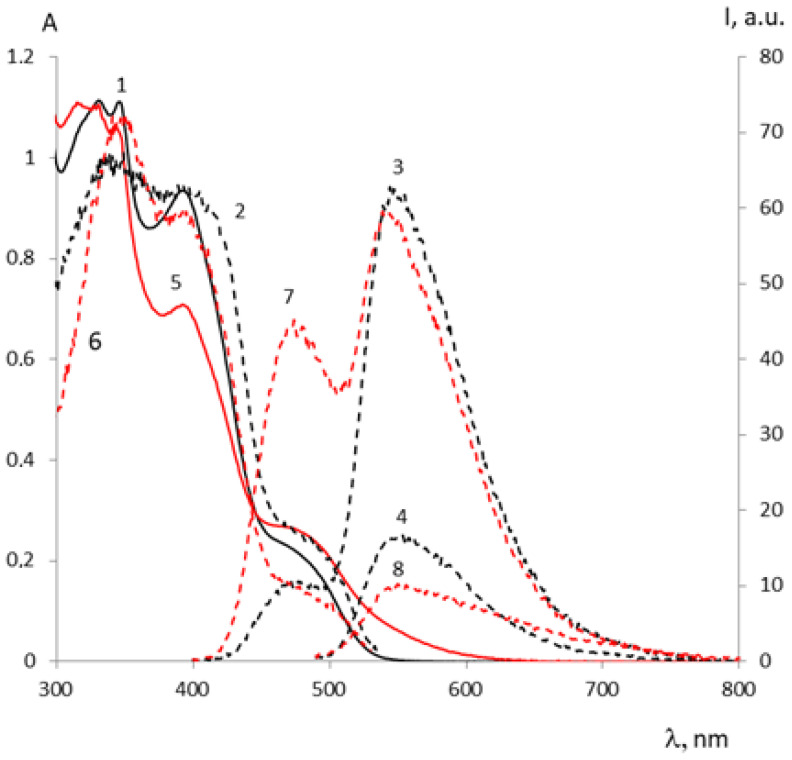
Absorption spectra (1, 5), fluorescence excitation spectra at λ_reg_ = 545 nm (2, 6) and fluorescence spectra at λ_ex_ = 390 nm (3, 7) and 480 nm (4, 8) of compound **1** before (1–4) and after UV irradiation by the external source (5–8) in the presence of Mg^2+^ ions in the mixed solvent solution.

The sharp increase in the **A^Kc^** content was observed immediately after Mg^2+^ ions introduction into the solution (Figure 6, spectrum 1) in comparison with the solution without metal ions (Figure 6). Under UV irradiation, the **A^Kc^** content decreased slightly with the simultaneous appearance of the photoinduced absorption of form **B** in the long-wavelength spectral region.

**Scheme 14:**
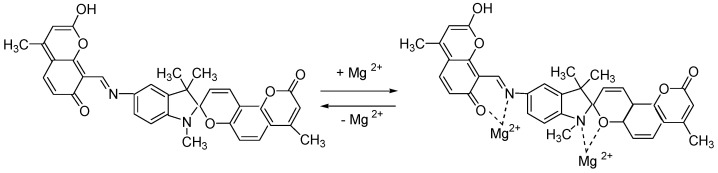
Assumed type of complex formation for the tautomeric form **A^T^** of compound **1** with Mg^2+^ ions.

Both fluorescence and fluorescence excitation spectra of the compound **1** solution in the presence of Mg^2+^ ions are identical to the spectra of the model compound **3** in the presence of Mg^2+^ ions (Figure 3) and **3** in ethanol solution (Figure 4). In the case of compound **1** and Mg^2+^ ions, Job’s method was used to determine the complex composition (Figure S9). The maximum of fluorescence at 475 nm was observed when the molar fraction reached 0.3, indicating 1:2 stoichiometry of complexation between **1** and Mg^2+^ ions. Consequently, the interaction of the closed spirocyclic form **A** of compound **1** with Mg^2+^ ions occurs similar to the model compounds **2** and **3**, namely at the azomethine part of the molecule and N-C_spiro_-O fragment (Scheme 14).

The assumption of the existence of two tautomeric forms of coumarin explains the fact that at the ratio compound **1**:metal ion = 1:100, complexation with the azomethinocoumarin fragment in the initial form **A** occurs only with a part of the molecules, probably with that portion that exists in the modified tautomeric form **A^T^**.

Two fluorescence bands with maxima at 475 nm and 545 nm are observed for hybrid compound **1** in the presence of Mg^2+^ ions when excited with λ_ex_ = 390 nm in the mixture of solvents (Figure 6, spectrum 3). The first of them is similar to the fluorescence of compound **3** complex with Mg^2+^, whereas the second band is assigned to the fluorescence of compound **3** caused by the ESIPT process.

Only one fluorescence with a maximum at 545 nm was observed when excited by λ_ex_ = 480 nm (Figure 6, spectrum 4) that is, at direct excitation of the *cis*-keto form. After UV irradiation of compound **1** solution containing Mg^2+^ ions, the intensity of the short-wavelength luminescence band increased sharply (Figure 6, spectrum 7). A similar phenomenon was observed earlier for ethanol solution of compound **1** [16]. A possible explanation for this phenomenon is that under the influence of UV irradiation the concentration of the tautomeric structure **T** of coumarin fragment increases, and only this **T**-form creates associates with ethanol and complexes with Mg^2+^.

**Figure 7:**
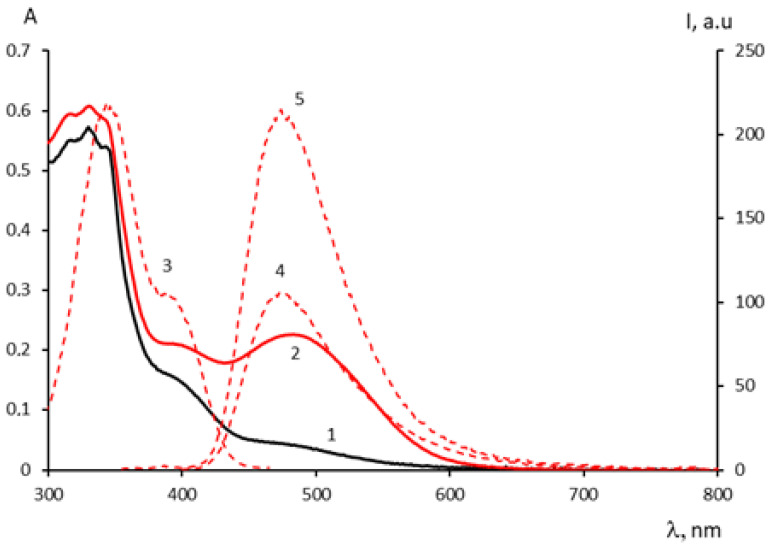
Absorption spectra (1, 2), fluorescence excitation spectrum at λ_reg_ = 475 nm (3) and fluorescence spectra at λ_ex_ = 390 nm (4) and 345 nm (5) before (1) and after UV irradiation (2–5) of compound **1** in the mixed solvent solution preliminary UV irradiated, kept in the dark and finally with Mg^2+^ ions added.

During the study of the behavior of compound **1** in the presence of Mg^2+^ ions, it was also discovered that when Mg(ClO_4_)_2_ solution is added to the solution of **1**, being preliminary UV irradiated and then kept in the dark, only one short-wavelength emission with a maximum at 475 nm and high intensity was observed after repeated UV irradiation (Figure 7, spectrum 5). This can be explained by the fact that under double UV irradiation, the amount of the tautomeric form **T** of the coumarin fragment sharply increased and, as a result, the concentration of its complex with Mg^2+^ ions also increased. Apparently, in this case all the molecules of the compound formed complexes with Mg^2+^ ions, in which ESIPT cannot be realized and in this case the long-wavelength band of fluorescence disappeared.

When Zn^2+^ ions were introduced into the mixed solvent solution of compound **1**, we observed phenomena similar to those occurring when Mg^2+^ ions were added, but less intense (see Figure S7). Here we can again assume the complexation mechanism with the tautomeric form **T**, similar to that assumed with Mg^2+^ ions.

## 3. Materials and Methods

### 3.1. Chemicals and Instrumentation

Toluene (99.8%, anhydrous) and acetonitrile (99.8%, anhydrous) were purchased from Aldrich (St. Louis, MO, USA) and were used as received.

The spectrophotometric studies in solutions were performed at ambient temperature on a Carry 50 bio spectrophotometer (Varian) in quartz cuvettes with 10 mm optical path length. For better dissolution of substances, the solutions were treated in a Sapphire ultrasonic bath for 10 min. The working concentration of the solution was 2 × 10^−4^ M in the presence of metal cations in the ratio [C]_comp_:[C]_Me_ = 1:100.

The fluorescence spectra were recorded on a CARY Eclipse (Varian B.V., Middelburg, The Netherlands) spectrofluorometer in a quartz cuvette with 10 mm optical path length. The fluorescence excitation spectra were also measured, which in all cases confirmed that they belonged to the compounds under study. The working concentration of the solution was 4 × 10^−5^ M in the presence of metal cations in the ratio [C]_comp_:[C]_Me_ = 1:100.

All spectral studies were performed in mixed toluene-acetonitrile (1:1 by volume) system. All salt were dissolved in acetonitrile and then added to toluene solutions of compounds under study.

Solutions were irradiated with light from an external source of an L8253 high intensity xenon lamp, which was a part of an LC-4 irradiator (Hamamatsu Photonics, Shizuoka, Japan), through a permeable to UV radiation UFS-1 colored glass filter (≈52.2 W/m^2^ in the range of 235–400 nm and 2.7 W/m^2^ in the visible range) for solution coloring, and through SZS-22 glass filter absorbing UV radiation and transmitting visible light for solution bleaching.

Hybrid functionals B3LYP and CAM-B3LYP and basis sets 6-311+G, Lanl2DZ were used for the calculations [35]. The calculations were carried out by Gaussian’09W software package. The modeling was carried out by PCM method using the integral equation formalism (IEFPCM) [36].

### 3.2. Synthesis

Synthesis of the hybrid compound 5′-[(7-hydroxy-4-methyl-2-oxo-3H-1-benzopyran-8-yl]-1′,3′-dihydro-1′,3′,3′,4-tetramethylspiro[[*2H*,*8H*]benzo[1,2-b,3,4-b’]dipyran-8,2′-[*2H*]indole]-2-one] **1** and the model 7-hydroxy-8-(4-methoxyphenyliminomethyl)-4-methyl-1-benzopyran-2-one) **3** was described earlier [16]. 1′,3′-Dihydro-1′,3′,3′,4-tetramethylspiro[[*2H*,*8H*]benzo[1,2-b,3,4-b’]dipyran-8,2′-[*2H*]indole-2-one] **2** was synthesized according to reference [24]. The 7-Hydroxy-4-methyl-1-benzopyran-2-one **3** (purity 97%) and LiClO_4_, Mg(ClO_4_)_2_∙6·H_2_O_,_ Ca(ClO_4_)_2_∙4·H_2_O, Zn(ClO_4_)_2_ ∙6·H_2_O were purchased from Aldrich.

## 4. Conclusions

Thus, two excited state proton transfers can occur in the model azomethinocoumarin **3** and hybrid compound **1**—the ESIPT of a proton from the OH group of 7-hydroxy coumarin tautomer to the N atom of the C=N bond of azomethine fragment and from the OH group of 7-hydroxy coumarin tautomer to the carbonyl group of the pyrone chromophore, which leads to the formation of the 2-hydroxyl-tautomer **T** of coumarin. The ESIPT occurs during UV excitation and leads to the appearance of fluorescence with a maximum at 550 nm. The second proton transfer takes place when the compound is irradiated with an external UV source, as well as upon complexation with ethanol molecules and a number of metals of the second group; it appears in the form of a shorter wavelength luminescence with a maximum at 470 nm. The dependence of fluorescence on the nature of metal ions and on the excitation wavelength was observed.

## Figures and Tables

**Table 1:** Spectral characteristics of the model compound **3** in the toluene/acetonitrile solution with and without metal ions.

Metal	λ_A_^max^, nm (D_A_^max^)	λ_ex.fl_, nm	λ_fl_^max^, nm before UV (I_fl_^max^, a.u.)	λ_fl_^max^, nm after UV (I_fl_^max^, a.u.)
-	338 (0.88), 380 (0.41 sh), 457 (0.02)	338	545 (36)	545 (31)
Li^+^	339 (0.81), 379 (0.3 sh), 461 (0.02)	338	548 (36)	548 (31)
Ca^2+^	339 (0.92), 382 (0.41 sh), 459 (0.04)	338	545 (36)	545 (33)
Zn^2+^	317 (0.49), 341 (0.48), 457 (0.02)	338	485 (29 sh)	485 (25 sh)
		338	528 (39)	528 (35)
Mg^2+^	338 (0.82), 386 (0.30 sh), 463 (0.03)	338	477 (41) 534 (50)	477 (77) 528 (62 sh)
		399	477 (2) 528 (20 sh)	477 (42) 528 (35)

λ_A_^max^, λ_ex.fl_ and λ_fl_^max^ are wavelengths for maxima of the absorption, fluorescence excitation and fluorescence emission bands, correspondingly; D_A_^max^ is the absorption in the maximum of the absorption band; I_fl_^max^ is the fluorescence intensity in the maximum of fluorescence band; sh is shoulder.

**Table 2:** Spectral characteristics of compound **1** and its complexes with metal ions in the mixed solvent solution at the ratio of molar concentrations of compound **1** and metal ion = 1: 100.

	λ_A_^max^, nm (D_A_^max^)	λ_B_ ^max^, nm	D_B_^phot^	± Δλ_B_, nm
-	330 (1.08), 345 (1.08), 390 (0.97), 480 (0.07 sh)	600	0.11	-
Li^+^	330 (1.10), 345 (1.10), 390 (0.99), 480 (0.07 sh)	580	0.01	−20
Ca^2+^	330 (1.13), 345 (1.15), 390 (0.93), 460 (0.29 sh)	560	0.02	−40
Mg^2+^	330 (1.10), 345 (1.10), 390 (0.93), 480 (0.23 sh)	540	0.07	−60

λ_A_^max^ and λ_B_^max^–are wavelengths for maxima of the absorption bands of **A** and **B** forms, correspondingly; D_A_^max^ is the absorption in the maximum of the absorption band; D_B_^phot^–is a change in the optical density at the maximum of the absorption band in the visible region of the **B** form spectrum; ± Δλ_B_–is a shift of the absorption band maximum of photoinduced form **B** in the presence of metal ions relative to the solution without ions.

**Table 3:** Fluorescence characteristics of compound **1** and its complexes with metal ions in the toluene/acetonitrile solution at the ratio between molar concentrations between compound (**1**) and metal ion = 1:100.

Metal	λ_1_^fl.max^, nm	I_1_^fl.max^, a. u.	λ_2_^fl.max^, nm	I_2_^fl.max^, a.u.
-	550	61	550	46
Li^+^	550	59	550	56
Ca^2+^	550	55	550	53
Zn^2+^	545	57	490 sh 545	23 35
Mg^2+^	475 545	10 63	475 540	45 59

λ_1_^fl.max^ and λ_2_^fl.max^—are wavelengths for maxima of fluorescence emission bands before and after UV irradiation; I_1_^fl.max^ and I_2_^fl.max^ are fluorescence intensities at the maximum of the fluorescence band before and after UV irradiation, respectively.

## Data Availability

Not applicable.

## Supplementary Materials

The following are available online, Figure S1: Kinetics of photo-coloring through a UFS-1 colored glass filter (235–400 nm) and dark relaxation of a solution of compound **2** in a mixture of toluene:acetonitrile solvents at the wavelength of 590 nm, Table S1: Spectral characteristics of spiropyran **2** and its complexes with metal ions in mixed solvent with the ratio C_L_/C_Me_ = 1/100, Figure S2: Absorption spectra of compound **2** in a mixture of toluene:acetonitrile solvents in the presence of Li^+^ ions in a solution (C_L_/C_Me_ = 1/100) before (1), after UV-irradiation and subsequent dark relaxation (3–5), Figure S3: Optimized models of tautometric **T** (a) and E (b) forms of azomethinocoumarin complexes with Mg^2+^ ions, Figure S4: Absorption spectra (1, 4, 6), fluorescence excitation spectra at λ_reg_ = 530 nm (3) and fluorescence at λ_ex_ = 338 nm (2, 5, 7) of compound **3** with Zn^2+^ ions in a mixture of toluene-acetonitrile solvents before (1–3) and after irradiation by UV (4, 5) and visible light (6, 7), Figure S5: Kinetics of fluorescence changes for **1** in a toluene solution at a wavelength of 545 nm (at λ_ex_ = 395 nm) during alternating irradiation with UV (down) and visible (up) light from an external source of high intensity, Figure S6: Absorption spectra (1, 5), fluorescence excitation spectra at λ_reg_ = 550 nm (2) and fluorescence at λ_ex_ = 390 nm (3, 6) and 490 nm (4, 7) before (1–4) and after UV irradiation (5–8) of compound **1** with Li^+^ ions in the mixed toluene:acetonitrile solution, Figure S7: Absorption spectra (1, 5), fluorescence excitation spectra at λ_reg_ = 545 nm (2, 6) and fluorescence at λ_ex_ = 390 nm (3, 7) and 460 nm (4, 8) before (1.4) and after UV-irradiation of compound **1** with Ca ^2+^ ions in the mixed toluene:acetonitrile solution, Figure S8: Absorption spectra (1, 5), fluorescence excitation spectra at λ_reg_ = 545 nm (2, 6) and fluorescence at λ_ex_ = 390 nm (3, 7) and 480 nm (4, 8) before (1–4) and after UV-irradiation compound **1** with Zn ^2+^ ions in the mixed toluene:acetonitrile solution, Figure S9: The changes in the fluorescence emission spectra at 475 nm of compound **1** at Mg^2+^addition.

## Sample Availability

Samples of the compounds under study are available from the authors.
