# Supplementary material for: Excited State Proton Transfers in Hybrid Compound Based on Indoline Spiropyran of the Coumarin Type and Azomethinocoumarin in the Presence of Metal Ions
Source: Molecules. 2021 Nov 16;26(22):6894. doi: 10.3390/molecules26226894 (PMC8624336; doi:10.3390/molecules26226894)
Supplement: Supplementary file 1 [file molecules-26-06894-s001.zip › molecules-1419839-supplementary.pdf]

Supplementary materials

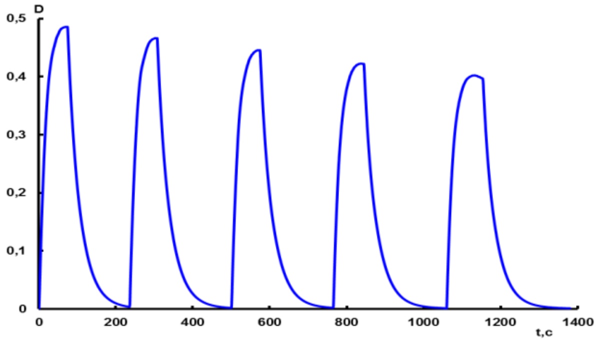

Figure S1. Kinetics of photo-coloring through a UFS-1 colored glass filter (235-400 nm) and dark relaxation of a solution of compound **2** in a mixture of toluene : acetonitrile solvents at the wavelength of 590 nm.

Table S1. Spectral characteristics of spiropyran **2** and its complexes with metal ions in mixed solvent with the ratio C_L/C_{Me} = 1/100

Ion	λ_A ^{max}, nm (D_A^{max})	λ_B ^{max}, nm	ΔD_B^{phot}	$\pm\Delta\lambda_B$, nm
-	332 (0.49), 345 (0.44)	405sh,		-
		590	0,48	
Li^+	332 (0.49), 345 (0.44)	405		
		560	0,17	-30
Ca^{2+}	332 (0.49), 345 (0.44)	405sh	0,02	-
		540	0,01	-50
Zn^{2+}	332 (0.59), 345 (0.53)	405	0,03	
		545	<0.01	-45
Mg^{2+}	332 (0.50), 345 (0.45)	405	0,02	-
		535	<0,01	-55

Note: λ_A ^{max} and λ_B ^{max} are the wavelengths of the maxima of the absorption bands of the initial and UV induced (in the visible spectra region) forms A and B, respectively; D_A^{max}– the optical density in the maximum of absorption band of initial form A, ΔD_B^{phot} – change in the optical density in the maximum of absorption band located in the visible region of the spectrum of photoinduced form B; $\pm\Delta\lambda_B$ – shift of the absorption band maximum of the photoinduced form B of spiropyran in the presence of metal ions relative to the solution

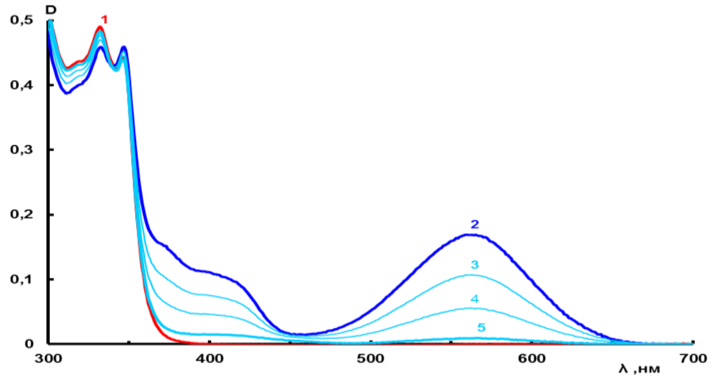

Figure S2. Absorption spectra of compound **2** in a mixture of toluene : acetonitrile solvents in the presence of Li^{+} ions in a solution (C_L/C_{Me} = 1/100) before (1), after UV - irradiation and subsequent dark relaxation (3-5).

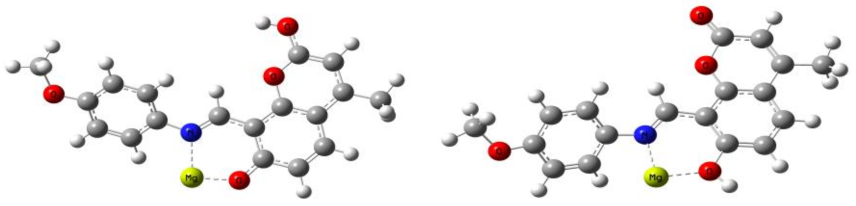

a b

Figure S3 . Optimized models of tautometric T (a) and E (b) forms of azomethinocoumarin complexes with Mg^{2+} ions.

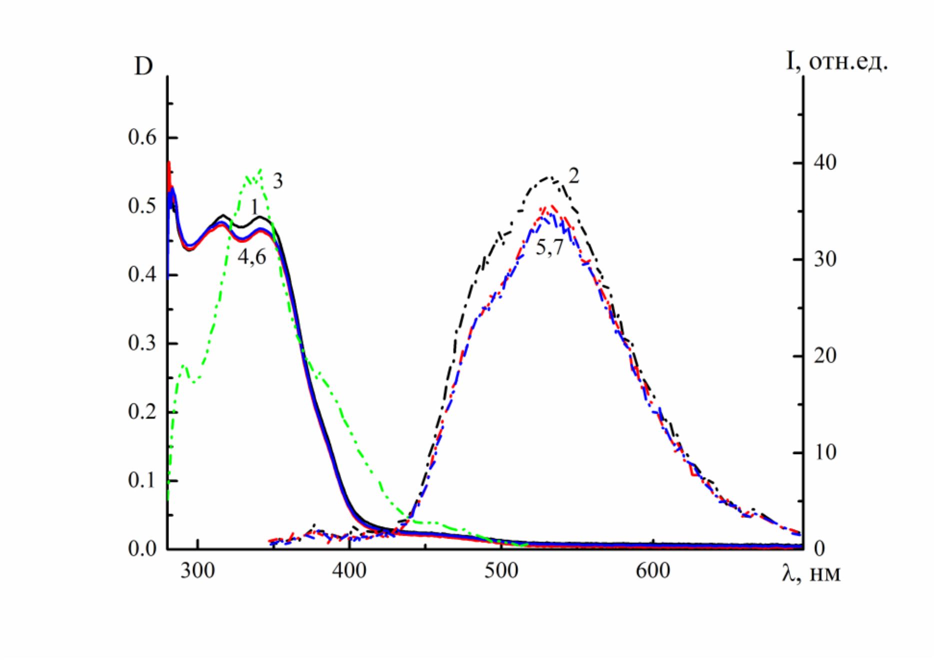

Figure S4. Absorption spectra (1, 4, 6), fluorescence excitation spectra at λ_{reg} = 530 nm (3) and fluorescence at λ_{ex} = 338 nm (2, 5, 7) of compound **3** with Zn^{2+} ions in a mixture of toluene-acetonitrile solvents before (1-3) and after irradiation by UV (4, 5) and visible light (6, 7).

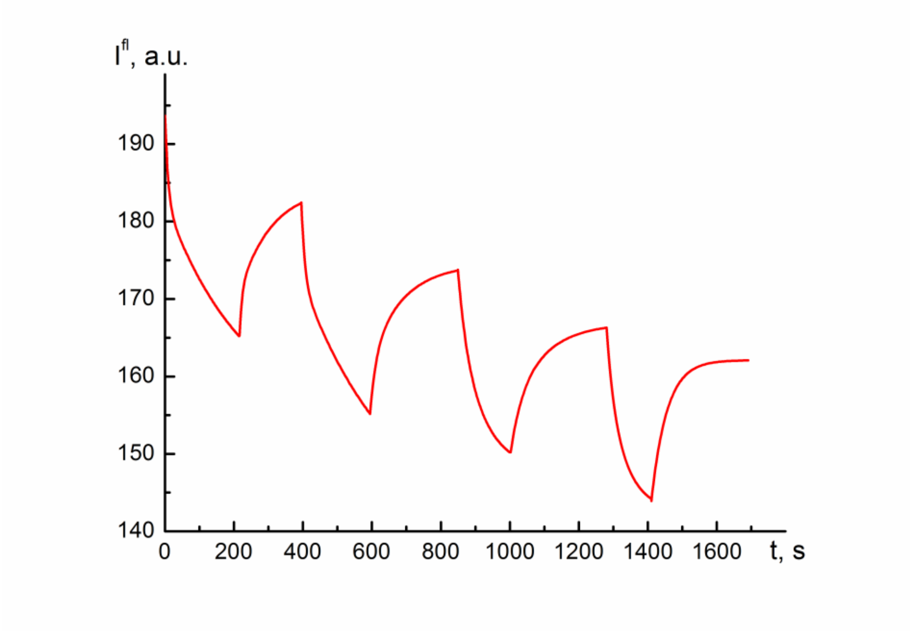

Figure S5. Kinetics of fluorescence changes for **1** in a toluene solution at a wavelength of 545 nm (at λ_{ex} = 395 nm) during alternating irradiation with UV (down) and visible (up) light from an external source of high intensity

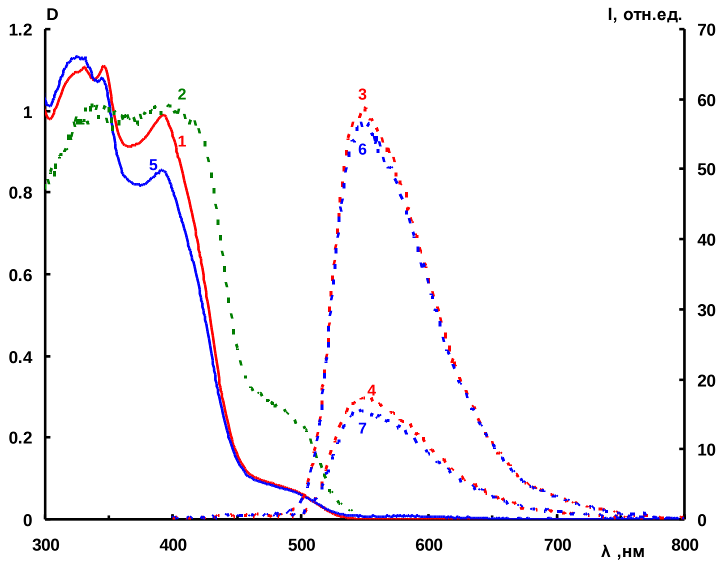

Figure S6. Absorption spectra (1,5), fluorescence excitation spectra at λ_{reg} = 550 nm (2) and fluorescence at λ_{ex} = 390 nm (3,6) and 490 nm (4, 7) before (1-4) and after UV irradiation (5-8) of compound **1** with Li^{+} ions in the mixed toluene : acetonitrile solution

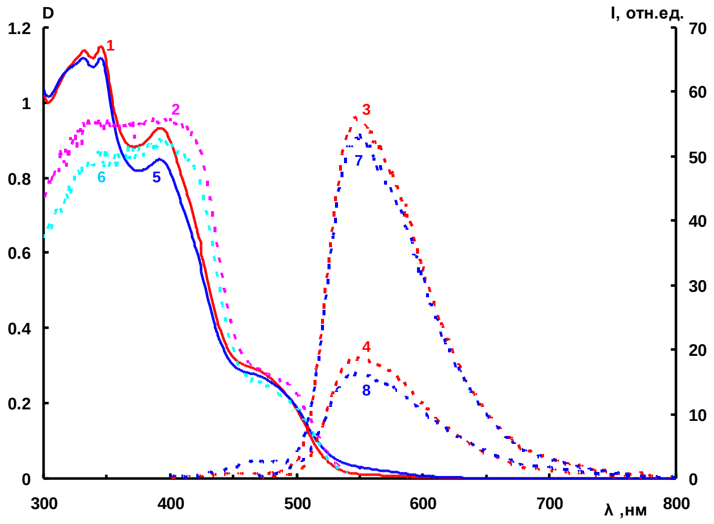

Figure S7. Absorption spectra (1,5), fluorescence excitation spectra at λ_{reg} = 545 nm (2, 6)) and fluorescence at λ_{ex} = 390 nm (3, 7) and 460 nm (4, 8) before (1.4) and after UV- irradiation of compound **1** with Ca^{2+} ions in the mixed toluene : acetonitrile solution.

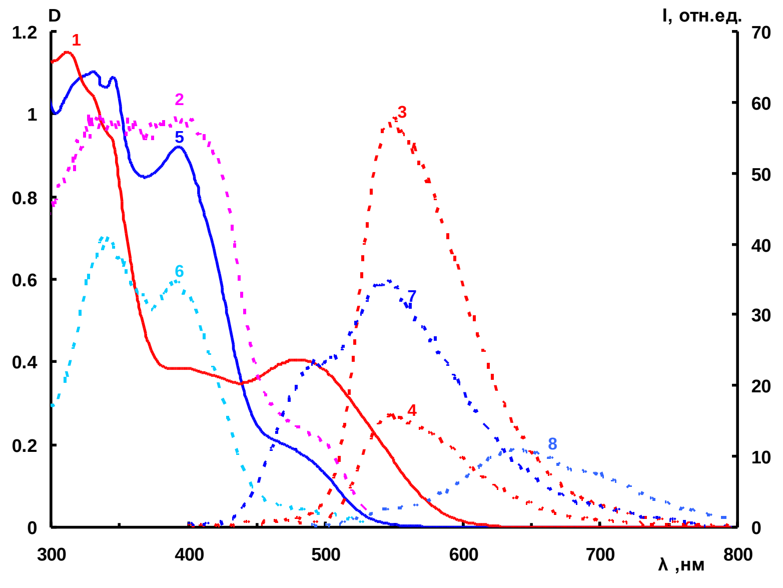

Figure S8. Absorption spectra (1,5), fluorescence excitation spectra at λ_{reg} =545 nm (2, 6) and fluorescence at λ_{ex} = 390 nm (3,7) and 480 nm (4,8) before (1-4) and after UV – irradiation compound **1** with Zn^{2+} ions in the mixed toluene : acetonitrile solution.

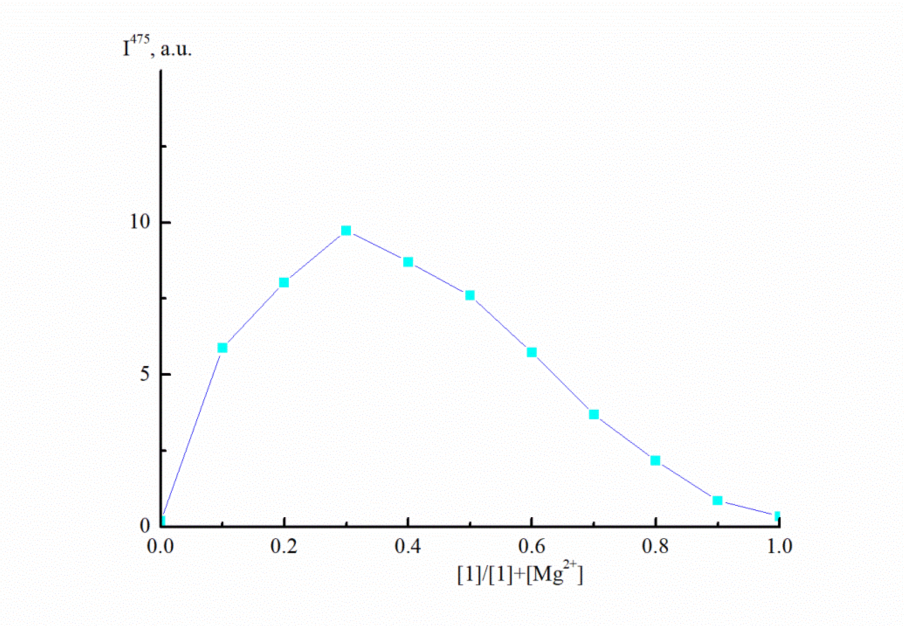

Figure S9. The changes in the fluorescence emission spectra at 475 nm of compound **1** at Mg^{2+}addition.

.
